# Supplementary material for: Timing of Resource Addition Affects the Migration Behavior of Wood Decomposer Fungal Mycelia
Source: J Fungi (Basel). 2021 Aug 12;7(8):654. doi: 10.3390/jof7080654 (PMC8402142; doi:10.3390/jof7080654)
Supplement: Supplementary file 1 [file jof-07-00654-s001.zip › jof-1320447-supplementary.pdf]

Supplementary Figure S1

Time-series photo images of mycelium of *Pholiota brunnescens, Phanerochaete velutina*, and *Resinicium bicolor* grew from inoculum on non-sterile compressed soil in Petri dishes (576 cm^2) in early-baited and wait experiments. (I) indicates inoculum wood blocks. (B) indicates bait wood blocks. One side length of each wood block is 1.5 cm.

Supplementary Figure S2

Photo images of inoculum and bait wood blocks of *Pholiota brunnescens*, *Phonerochaete velutina*, and *Resinicium bicolor* 7 days after transferred onto a new soil dish in early-baited and wait experiments.

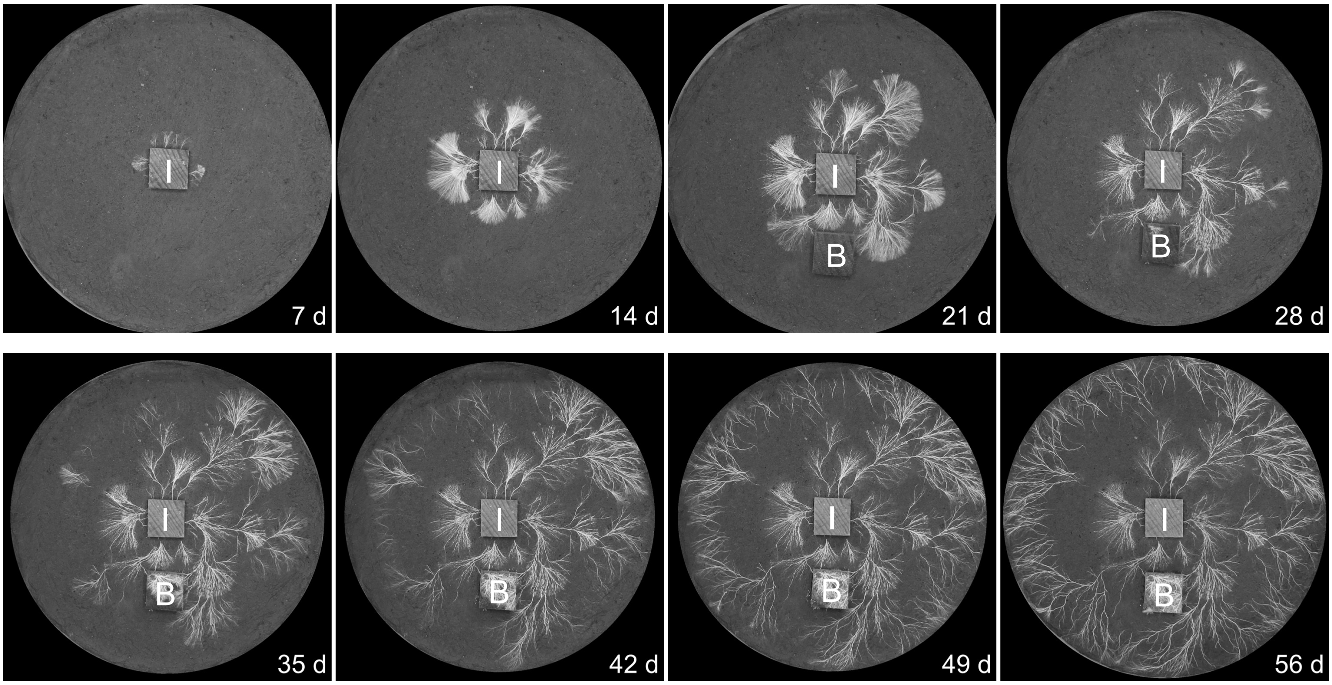

Fig. S1a Time-series photo images of mycelium of *Pholiota brunnescens* (Pb_B18) in early-baited experiment grew from inoculum (I). A fresh wood bait (B) was added at 14 d.

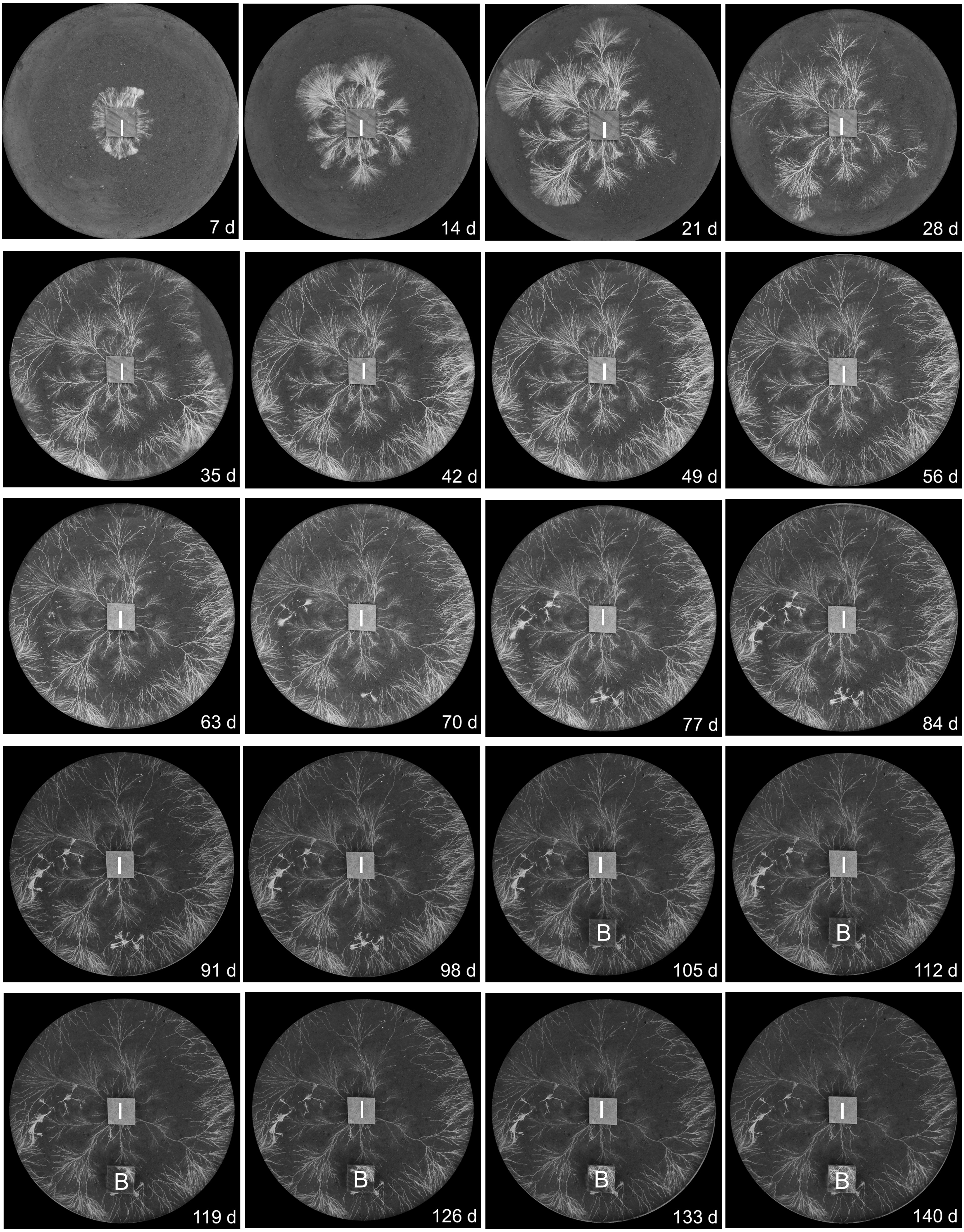

Fig. S1b Time-series photo images of mycelium of *Pholiota brunnescens* (Pb_B11) in wait experiment grew from inoculum (I). A fresh wood bait (B) was added at 98 d.

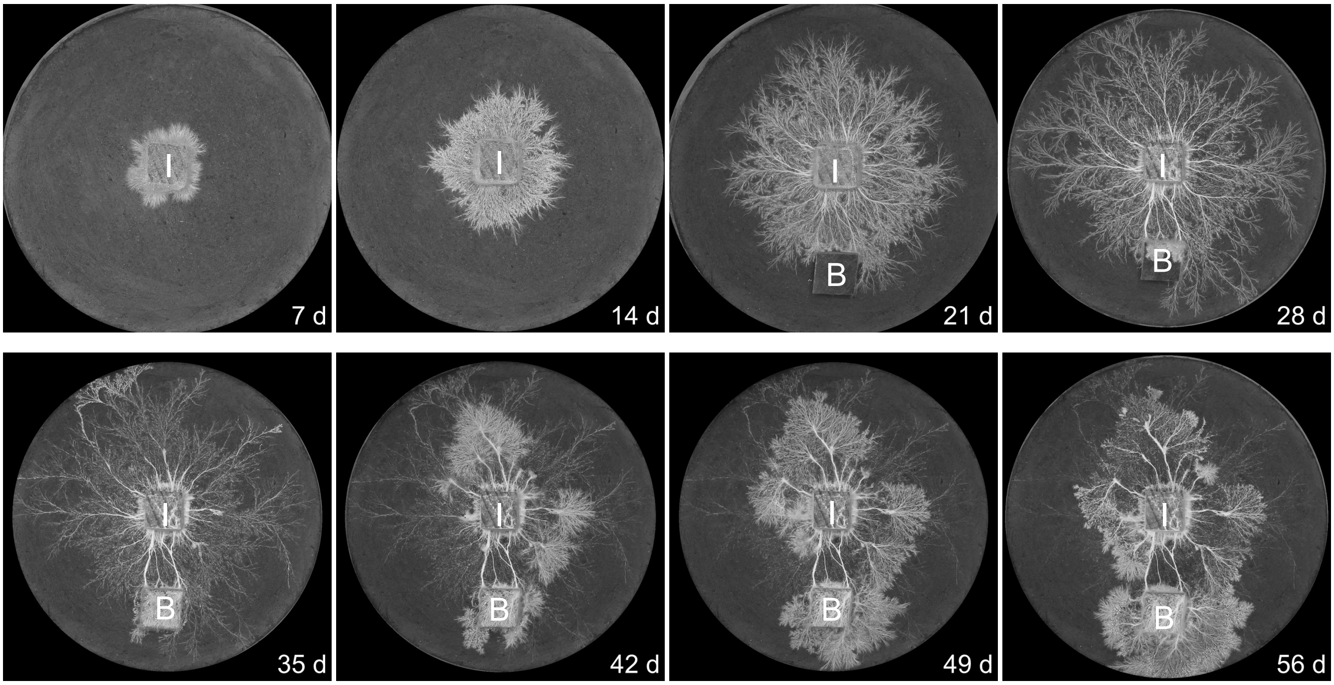

Fig. S1c Time-series photo images of mycelium of *Phanerocaete velutina* (Pv_B6) in early-baited experiment grew from inoculum (I). A fresh wood bait (B) was added at 98 d.

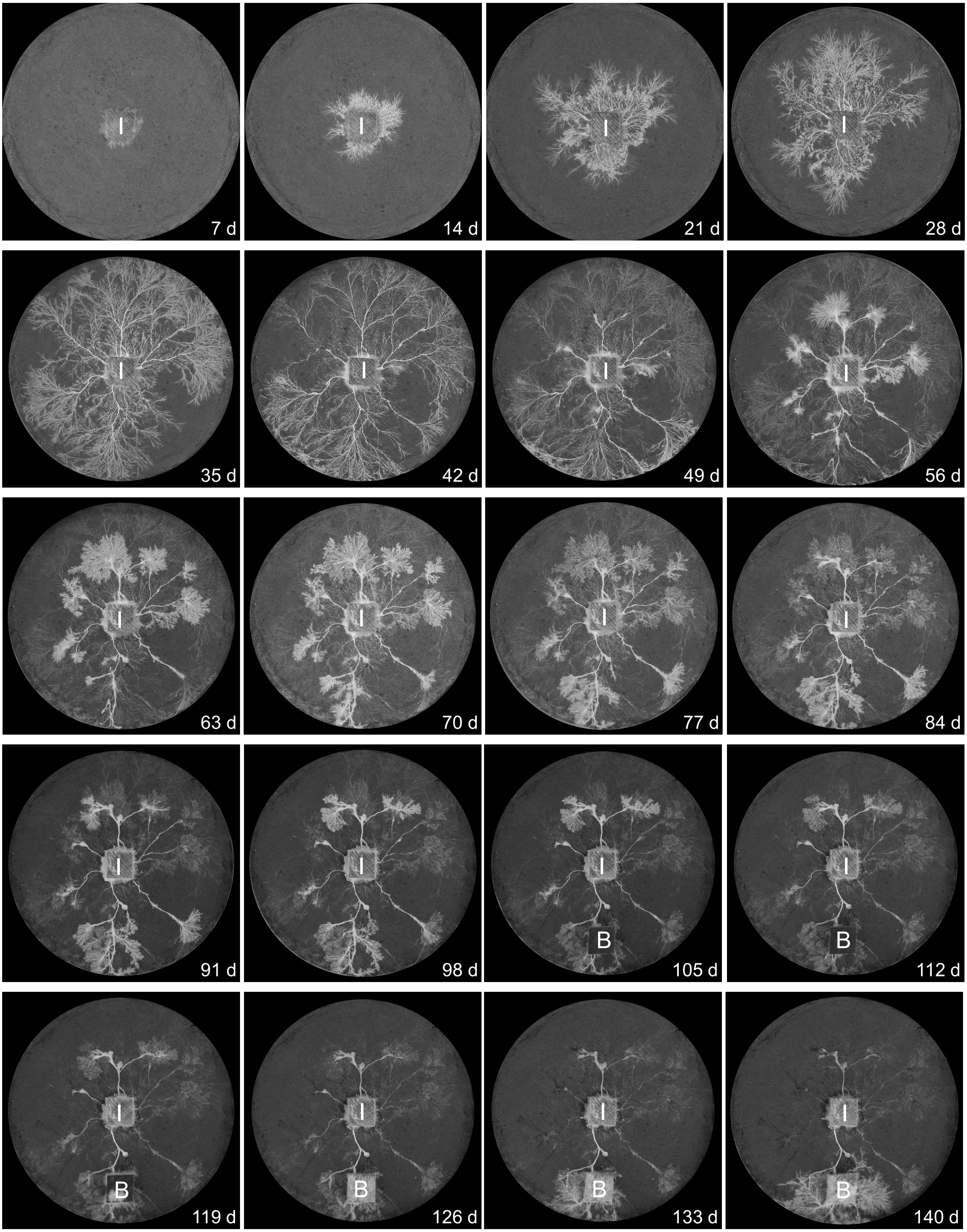

Fig. S1d Time-series photo images of ¥ mycelium of *Phanerocaete velutina* (Pv_B13) in wait experiment grew from inoculum (I). A fresh wood bait (B) was added at 98 d.

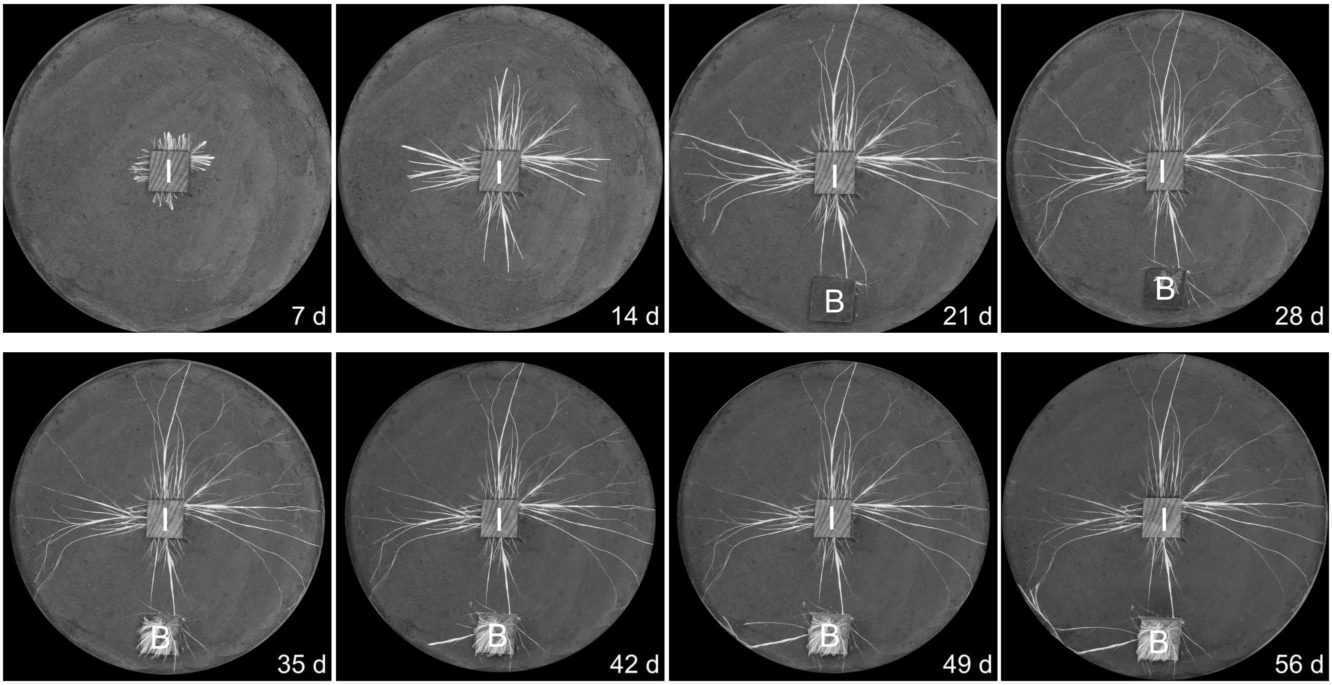

Fig. S1e Time-series photo images of mycelium of *Resinicium bicolor in* early-baited experiment grew from inoculum (I). A fresh wood bait (B) was added at 14 d.

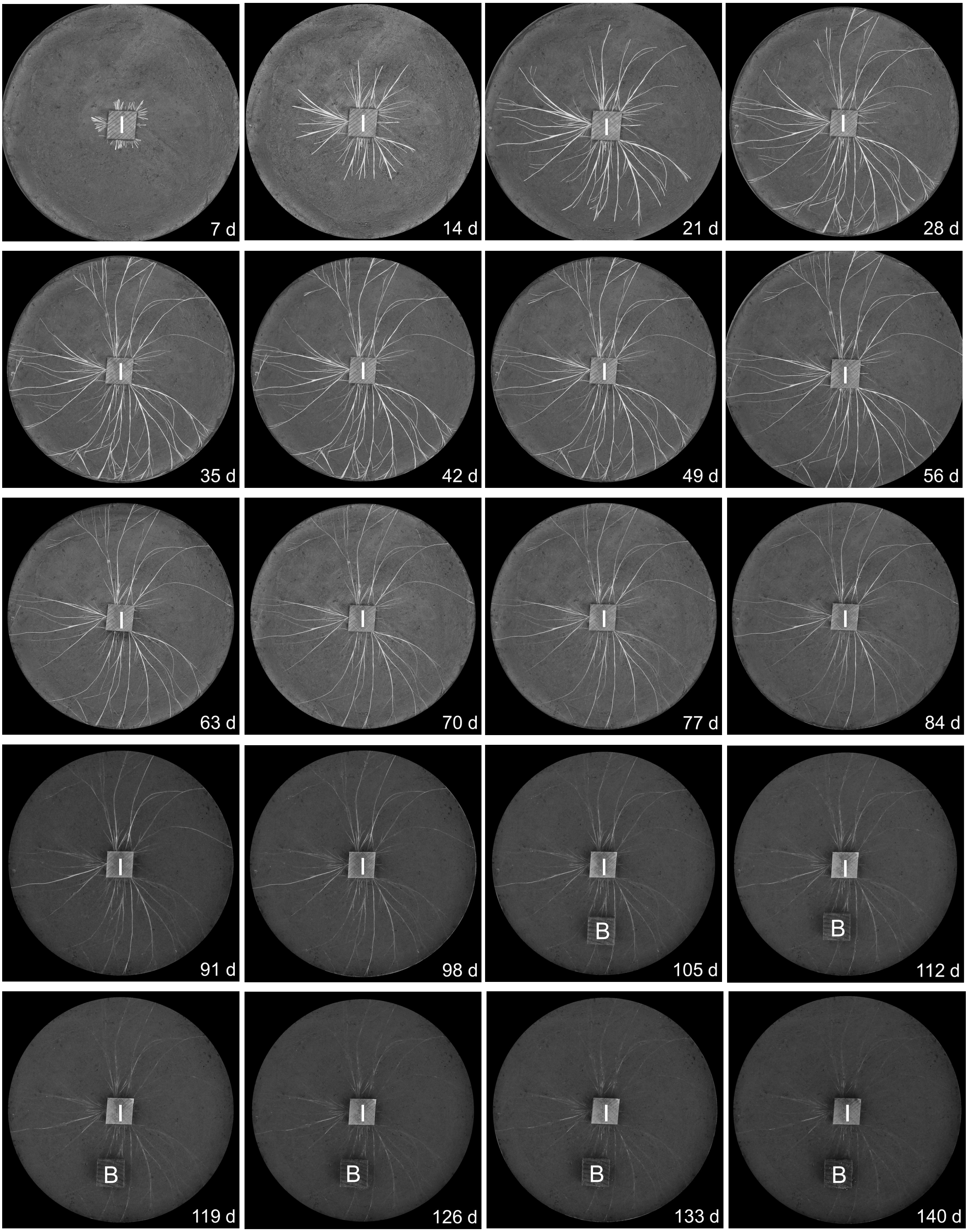

Fig. S1f Time-series photo images of mycelium of *Resinicium bicolor* in wait experiment grew from inoculum (I). A fresh wood bait (B) was added at 98 d.

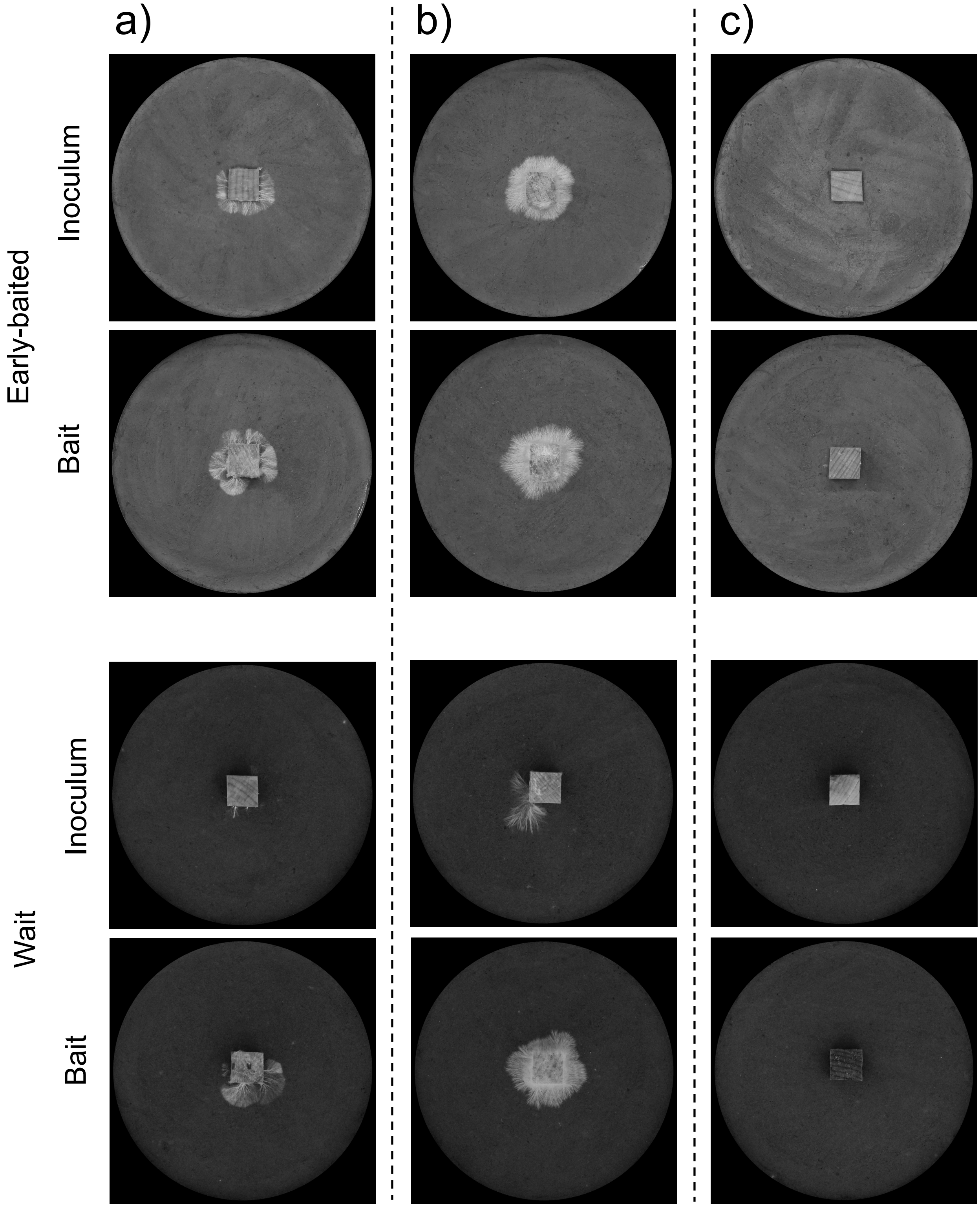

Fig. S2 Photo images of inoculum and bait wood blocks of a) *Pholiota brunnescens*, b) *Phonerochaete velutina*, and c) *Resinicium bicolor* 7 days after transferred onto a new soil dish in early-baited and wait experiments. Note that the relative position of inoculum and bait photos reflects their relative positions on soil before transfer (i.e., a bait located at bottom side of an inoculum, see Figs. 1 and Fig. S1).
